# Supplementary material for: Pitfalls in using ML to predict cognitive function performance
Source: Sci Rep. 2025 Oct 29;15:37747. doi: 10.1038/s41598-025-24325-9 (PMC12572133; doi:10.1038/s41598-025-24325-9)
Supplement: Supplementary file 1 — Supplementary Material 1 [file 41598_2025_24325_MOESM1_ESM.docx]

**Pitfalls in using ML to predict cognitive function performance**

**Appendix A**

| **Test** | **Abbreviation** | **Description** | **Variables** |
| --- | --- | --- | --- |
| COGNITIVE FLEXIBILITY | | | |
| Trail Making Test | TMT | The task consists of 2 parts. In part A, numbers from 1–25 are displayed on the screen in a haphazard fashion. The task consists of clicking on the numbers in sequential order as quickly as possible. In part B numbers from 1–13 and letters from A-L are presented on the screen. participants must click on the numbers and letters alternately and in ascending order. | Processing time part A, Processing time part B, Difference part B-A [seconds], Quotient B/A, Errors part A, Errors part B |
| Raven’s Standard Progressive Matrices | SPM | Eight items that form one pattern are shown to the participants. The task requires the participants to identify one missing item out of 6 choices to complete the pattern. The difficulty of recognizing each pattern increases during the course of this test. | Correct items, Processing time |
| Wisconsin Card Sorting Test | WCST | Four stimulus cards illustrating different geometrical figures are presented. These cards differ in the number, color and form of the figures. The task is to match one additional card to one of the four cards using the correct rule (match for number, colour or form of figure) without knowing which rule is applied.Thus, participants are required to shift rules accordingly. | Number of errors, Number of perseveration errors, Number of errors (non perseveration), Timeouts |
| Tower Of London | TOL | Three rods are presented on the screen: The left rod holds three balls, the middle rod two balls and the right rod one ball. The participants are asked to move the balls from the starting state to ta target position using a minumum number of moves. | Planning ability, Number of correct responses, Changed his/her mind, self-correction, Choice of wrong pole, Choice of blocked pole, Choice of impossible position |
| Cued Task Switching | SWITCH | A coloured figure is presented on the screen. Participants are required to respond to either the color or figure task. Figure task: Particpants press matching button (left or right) depending on the type of the figure (triangle or rectangle); colour task: Particpants press matching button (left or right) depending on the colour of the figure (blue or yellow). | Number of errors, Timeouts, Errors of items which are incongruent |
| WORKING MEMORY | | | |
| N-back Non-Verbal | NBN | A sequence of 100 abstract successive figures are presented to the particpants. The task consists of indicating whether the current stimulus matches the figure shown two turns back (2-back paradigm). | Correct items, Number of commission errors, Number of errors, Mean reaction tine of correct items [seconds], Mean reaction time of errors [seconds] |
| Non-Verbal Learning Test | NVLT | Nonsensical, irregular, and geometric figures are presented on the screen. In the course of the test some figures are shown multiple times. For each figure the participants has to decide whether the current figure has already appeared or whether this figure is being shown for the first time. | Sum of correct responses, Sum of false responses, Sum of difference between correct minus false responses, Processing time |
| Corsi Block Tapping Test | CORSI | Nine irregularly arranged cubes are presented to the participants. A cursor touches a certain number of cubes in a specific order; The task is to repeat the given sequence correctly. The length of the sequence increases the more correct sequences the particpants complete. | Block span, Correct items, False items, Missed items, Sequency errors |
| INHIBITION | | | |
| Stop Signal Task | INHIB | The test consists of two parts: 1) The participants are asked to respond to the direction of an arrow stimulus. 2) The participants have to repeat task as in previous step but should withhold their motoric response whenever they hear an auditory signal. | Reaction time [seconds], Mean stop signal delay [seconds], Stop signal reaction time [seconds], Number of commission errors, Number of ommission errors |
| Simon Task | SIMON | The participants are asked to press the right button if they read the word "right" and the left button if they read the word "left". The words are either presented on the right or left part of the screen. The reaction time of the participants is usually longer whenever the stimlus is incongruent to its position (e.g. the word "left" is on the right side of the screen). | Number of errors in compatible items, Number of errors in incompatible items |
| Stroop Test | STROOP | Names of colors (e.g., "blue", "green", or "red") are displayed on the screen in a color which is not denoted by the name (i.e., the word "blue" is printed in red). The test consists of two conditions: 1) Naming - participants are asked to respond to the colour of the words; 2) Reading - participants are asked to respond to the meaning of the word with naming. A baseline measure is taken at the start of the test to assess reading and color naming (color and word refer to the same concept). | Reading interference [seconds], Naming interference [seconds], Interference-difference [seconds], Number of false reactions (reading-baseline), Number of false reactions (naming-baseline), Number of false reactions (reading-interference), Number of false reactions (naming-interference), Processing time |
| ATTENTION / VIGILANCE | | | |
| Divided Attention Test | WAF-G | The participants are required to focus on two geometric figures and one auditory stimulus. At a certain interval the stimuli change their intensitiy (figure gets lighter and/ or auditory stimulus gets higher). The participants have to respond when two stimuli become lighter/higher twice in a row. | Number of missed items (unimodall visual), Number of false alarm (unimodal visual), Mean reaction time (unimodal visual) [ms], Number of missed items (crossmodal visual/auditive), Number of false alarm (crossmodal visual/auditive), Mean reaction time (crossmodal) [ms] |
| Spatial Attention Test | WAF-R | Four triangles are presented in four spatial positions (similar to Posner paradigm). The participants are required to react if a triangle changes intensity (gets darker). In the neglect test a interfering/matching visual cue is given but this cue do not always indicate the correct answer. | Mean reaction time (unannounced items) [ms], Number of missed items (correct announced items), Mean reaction time (correct announced items) [ms], Number of missed items (wrong announced items), Mean reaction time (wrong announced items) [ms], Mean reaction time (short SOA) [ms], Mean reaction time (long SOA) [ms], Number of errors |
| Mackworth Clock Test | MACK | The device has a large black pointer in a large circular background like a clock. The pointer moves in short jumps like the second hand of an analog clock, approximately every second. At infrequent and irregular intervals, the hand makes a double jump, e.g. 12 times every 30 seconds. The task is to detect when the double jumps occur by pressing a button. | Number of missed jumps, Number of false alarms |

**Table S1.** Assessed executive function variables with descriptions. Adapted from Amunts et al., 2020; 2021.

**Appendix B**

|  | **variable** | **importance** |
| --- | --- | --- |
| **0** | F0semitoneFrom27.5Hz sma3nz amean x | 0.0007097025948518670 |
| **1** | F0semitoneFrom27.5Hz sma3nz stddevNorm x | 0.0028338968486788300 |
| **2** | F0semitoneFrom27.5Hz sma3nz percentile20.0 x | 0.001696543627845140 |
| **3** | F0semitoneFrom27.5Hz sma3nz percentile50.0 x | 0.002228309253987480 |
| **4** | F0semitoneFrom27.5Hz sma3nz percentile80.0 x | 0.002052423004310750 |
| **5** | F0semitoneFrom27.5Hz sma3nz pctlrange0-2 x | 0.0020258769458956600 |
| **6** | F0semitoneFrom27.5Hz sma3nz meanRisingSlope x | 0.0021765621566742100 |
| **7** | F0semitoneFrom27.5Hz sma3nz stddevRisingSlope x | 0.004961085533184740 |
| **8** | F0semitoneFrom27.5Hz sma3nz meanFallingSlope x | 0.0005978817413192580 |
| **9** | F0semitoneFrom27.5Hz sma3nz stddevFallingSlope x | 0.002355452137794320 |
| **10** | loudness sma3 amean x | 0.0018408754710885800 |
| **11** | loudness sma3 stddevNorm x | 0.0025773925758115500 |
| **12** | loudness sma3 percentile20.0 x | 0.0026731443585815900 |
| **13** | loudness sma3 percentile50.0 x | 0.0012851617258609900 |
| **14** | loudness sma3 percentile80.0 x | 0.001131255155431440 |
| **15** | loudness sma3 pctlrange0-2 x | 0.0004220055730922390 |
| **16** | loudness sma3 meanRisingSlope x | 0.000556607092032695 |
| **17** | loudness sma3 stddevRisingSlope x | 0.0038216802193669900 |
| **18** | loudness sma3 meanFallingSlope x | 0.0012033523001281900 |
| **19** | loudness sma3 stddevFallingSlope x | 0.0007120468242543740 |
| **20** | spectralFlux sma3 amean x | 0.0010964926120670000 |
| **21** | spectralFlux sma3 stddevNorm x | 0.004786547677963480 |
| **22** | mfcc1 sma3 amean x | 0.0002515328126387560 |
| **23** | mfcc1 sma3 stddevNorm x | 0.006029604919235870 |
| **24** | mfcc2 sma3 amean x | 0.0010989626393870400 |
| **25** | mfcc2 sma3 stddevNorm x | 0.005563551732154450 |
| **26** | mfcc3 sma3 amean x | 0.0015540319512514700 |
| **27** | mfcc3 sma3 stddevNorm x | 0.0077412814247611900 |
| **28** | mfcc4 sma3 amean x | 0.005628298620739790 |
| **29** | mfcc4 sma3 stddevNorm x | 0.003958633619521410 |
| **30** | jitterLocal sma3nz amean x | 0.0017195260905201300 |
| **31** | jitterLocal sma3nz stddevNorm x | 0.001529678053734570 |
| **32** | shimmerLocaldB sma3nz amean x | 0.004133283355080180 |
| **33** | shimmerLocaldB sma3nz stddevNorm x | 0.0028863215767445700 |
| **34** | HNRdBACF sma3nz amean x | 0.0010284199580008700 |
| **35** | HNRdBACF sma3nz stddevNorm x | 0.0009677714997704380 |
| **36** | logRelF0-H1-H2 sma3nz amean x | 0.002478934734301150 |
| **37** | logRelF0-H1-H2 sma3nz stddevNorm x | 0.0013191197263624600 |
| **38** | logRelF0-H1-A3 sma3nz amean x | 0.0011657699928674900 |
| **39** | logRelF0-H1-A3 sma3nz stddevNorm x | 0.0018147620508413200 |
| **40** | F1frequency sma3nz amean x | 0.002008634759764940 |
| **41** | F1frequency sma3nz stddevNorm x | 0.0033652610787596800 |
| **42** | F1bandwidth sma3nz amean x | 0.002000803719123280 |
| **43** | F1bandwidth sma3nz stddevNorm x | 0.00993160375939468 |
| **44** | F1amplitudeLogRelF0 sma3nz amean x | 0.00030460761429602200 |
| **45** | F1amplitudeLogRelF0 sma3nz stddevNorm x | 0.000847348214375289 |
| **46** | F2frequency sma3nz amean x | 0.0012472907120759200 |
| **47** | F2frequency sma3nz stddevNorm x | 0.005366653032348730 |
| **48** | F2bandwidth sma3nz amean x | 0.00465349658853416 |
| **49** | F2bandwidth sma3nz stddevNorm x | 0.0017179786348170300 |
| **50** | F2amplitudeLogRelF0 sma3nz amean x | 0.0005301024117664150 |
| **51** | F2amplitudeLogRelF0 sma3nz stddevNorm x | 0.0011607105617351700 |
| **52** | F3frequency sma3nz amean x | 0.002299230696545290 |
| **53** | F3frequency sma3nz stddevNorm x | 0.002341285395649140 |
| **54** | F3bandwidth sma3nz amean x | 0.009952911694038720 |
| **55** | F3bandwidth sma3nz stddevNorm x | 0.003240888630236150 |
| **56** | F3amplitudeLogRelF0 sma3nz amean x | 0.0020590343652762000 |
| **57** | F3amplitudeLogRelF0 sma3nz stddevNorm x | 0.0018296644390162400 |
| **58** | alphaRatioV sma3nz amean x | 0.005110412116013940 |
| **59** | alphaRatioV sma3nz stddevNorm x | 0.00422340248676144 |
| **60** | hammarbergIndexV sma3nz amean x | 0.003264165687400150 |
| **61** | hammarbergIndexV sma3nz stddevNorm x | 0.0032688628796598600 |
| **62** | slopeV0-500 sma3nz amean x | 0.0016727259736228300 |
| **63** | slopeV0-500 sma3nz stddevNorm x | 0.005313853132453070 |
| **64** | slopeV500-1500 sma3nz amean x | 0.0029247031385025400 |
| **65** | slopeV500-1500 sma3nz stddevNorm x | 0.0169507039377196 |
| **66** | spectralFluxV sma3nz amean x | 0.0007108261466034310 |
| **67** | spectralFluxV sma3nz stddevNorm x | 0.0066760903887396500 |
| **68** | mfcc1V sma3nz amean x | 0.0025661831952822000 |
| **69** | mfcc1V sma3nz stddevNorm x | 0.0026017759087722900 |
| **70** | mfcc2V sma3nz amean x | 0.0014742673170024400 |
| **71** | mfcc2V sma3nz stddevNorm x | 0.0012015813027141900 |
| **72** | mfcc3V sma3nz amean x | 0.0032718406985272700 |
| **73** | mfcc3V sma3nz stddevNorm x | 0.0016587113724030200 |
| **74** | mfcc4V sma3nz amean x | 0.004330583686537030 |
| **75** | mfcc4V sma3nz stddevNorm x | 0.00964304238878294 |
| **76** | alphaRatioUV sma3nz amean x | 0.0017094783831153000 |
| **77** | hammarbergIndexUV sma3nz amean x | 0.0013367764067181700 |
| **78** | slopeUV0-500 sma3nz amean x | 0.0022581906477136100 |
| **79** | slopeUV500-1500 sma3nz amean x | 0.0022499778491361200 |
| **80** | spectralFluxUV sma3nz amean x | 0.0012905997268171500 |
| **81** | loudnessPeaksPerSec x | 0.0012162152706945800 |
| **82** | VoicedSegmentsPerSec x | 0.001217721890795340 |
| **83** | MeanVoicedSegmentLengthSec x | 0.0009672349097011730 |
| **84** | StddevVoicedSegmentLengthSec x | 0.0005381382305746130 |
| **85** | MeanUnvoicedSegmentLength x | 0.001147862329923420 |
| **86** | StddevUnvoicedSegmentLength x | 0.003826817309921350 |
| **87** | equivalentSoundLevel dBp x | 0.0011051463371811400 |
| **88** | F0semitoneFrom27.5Hz sma3nz amean y | 0.009296823138476110 |
| **89** | F0semitoneFrom27.5Hz sma3nz stddevNorm y | 0.0023551104143184300 |
| **90** | F0semitoneFrom27.5Hz sma3nz percentile20.0 y | 0.0036408272383118100 |
| **91** | F0semitoneFrom27.5Hz sma3nz percentile50.0 y | 0.0018716119993800200 |
| **92** | F0semitoneFrom27.5Hz sma3nz percentile80.0 y | 0.0037349464387791800 |
| **93** | F0semitoneFrom27.5Hz sma3nz pctlrange0-2 y | 0.004737214370203530 |
| **94** | F0semitoneFrom27.5Hz sma3nz meanRisingSlope y | 0.0028422162991930000 |
| **95** | F0semitoneFrom27.5Hz sma3nz stddevRisingSlope y | 0.0021561189186049000 |
| **96** | F0semitoneFrom27.5Hz sma3nz meanFallingSlope y | 0.004146546166840130 |
| **97** | F0semitoneFrom27.5Hz sma3nz stddevFallingSlope y | 0.0014815873525847400 |
| **98** | loudness sma3 amean y | 0.0015407748995659600 |
| **99** | loudness sma3 stddevNorm y | 0.002168392479517110 |
| **100** | loudness sma3 percentile20.0 y | 0.002362165184722420 |
| **101** | loudness sma3 percentile50.0 y | 0.0020153930747512800 |
| **102** | loudness sma3 percentile80.0 y | 0.003878075217373660 |
| **103** | loudness sma3 pctlrange0-2 y | 0.003667209111435230 |
| **104** | loudness sma3 meanRisingSlope y | 0.0012799312321853 |
| **105** | loudness sma3 stddevRisingSlope y | 0.004973605457607900 |
| **106** | loudness sma3 meanFallingSlope y | 0.00036927654856665300 |
| **107** | loudness sma3 stddevFallingSlope y | 0.001999821892991590 |
| **108** | spectralFlux sma3 amean y | 0.0017910721229046400 |
| **109** | spectralFlux sma3 stddevNorm y | 0.0011972496223693600 |
| **110** | mfcc1 sma3 amean y | 0.0007978220834346870 |
| **111** | mfcc1 sma3 stddevNorm y | 0.004295301515685850 |
| **112** | mfcc2 sma3 amean y | 0.00178558551936489 |
| **113** | mfcc2 sma3 stddevNorm y | 0.009947169838790750 |
| **114** | mfcc3 sma3 amean y | 0.0012718395994752600 |
| **115** | mfcc3 sma3 stddevNorm y | 0.03913224543093840 |
| **116** | mfcc4 sma3 amean y | 0.0036710681242518300 |
| **117** | mfcc4 sma3 stddevNorm y | 0.0052339436391217300 |
| **118** | jitterLocal sma3nz amean y | 0.002711402693269000 |
| **119** | jitterLocal sma3nz stddevNorm y | 0.0024519795823265500 |
| **120** | shimmerLocaldB sma3nz amean y | 0.0019858714373093000 |
| **121** | shimmerLocaldB sma3nz stddevNorm y | 0.0014751173848518700 |
| **122** | HNRdBACF sma3nz amean y | 0.003015895369353590 |
| **123** | HNRdBACF sma3nz stddevNorm y | 0.004306872893969350 |
| **124** | logRelF0-H1-H2 sma3nz amean y | 0.0022461980434457700 |
| **125** | logRelF0-H1-H2 sma3nz stddevNorm y | 0.006123553614387490 |
| **126** | logRelF0-H1-A3 sma3nz amean y | 0.0023603497271874400 |
| **127** | logRelF0-H1-A3 sma3nz stddevNorm y | 0.008926783877076220 |
| **128** | F1frequency sma3nz amean y | 0.0028242285923619500 |
| **129** | F1frequency sma3nz stddevNorm y | 0.0038701735122170800 |
| **130** | F1bandwidth sma3nz amean y | 0.002521831965706240 |
| **131** | F1bandwidth sma3nz stddevNorm y | 0.001311360740681930 |
| **132** | F1amplitudeLogRelF0 sma3nz amean y | 0.0016290004873711300 |
| **133** | F1amplitudeLogRelF0 sma3nz stddevNorm y | 0.0008943552238530020 |
| **134** | F2frequency sma3nz amean y | 0.001366359948825560 |
| **135** | F2frequency sma3nz stddevNorm y | 0.0036771647071178800 |
| **136** | F2bandwidth sma3nz amean y | 0.011425798833834400 |
| **137** | F2bandwidth sma3nz stddevNorm y | 0.0030375353343009500 |
| **138** | F2amplitudeLogRelF0 sma3nz amean y | 0.0009041315039012720 |
| **139** | F2amplitudeLogRelF0 sma3nz stddevNorm y | 0.00394588054386265 |
| **140** | F3frequency sma3nz amean y | 0.0018208068110509200 |
| **141** | F3frequency sma3nz stddevNorm y | 0.004531377741517610 |
| **142** | F3bandwidth sma3nz amean y | 0.0019560950986703900 |
| **143** | F3bandwidth sma3nz stddevNorm y | 0.003178119301889560 |
| **144** | F3amplitudeLogRelF0 sma3nz amean y | 0.0008519911219058000 |
| **145** | F3amplitudeLogRelF0 sma3nz stddevNorm y | 0.0059353918223872700 |
| **146** | alphaRatioV sma3nz amean y | 0.002138204542064240 |
| **147** | alphaRatioV sma3nz stddevNorm y | 0.01103339220642980 |
| **148** | hammarbergIndexV sma3nz amean y | 0.0008504120045999740 |
| **149** | hammarbergIndexV sma3nz stddevNorm y | 0.004958417989828240 |
| **150** | slopeV0-500 sma3nz amean y | 0.0012823343275550300 |
| **151** | slopeV0-500 sma3nz stddevNorm y | 0.004949941102124320 |
| **152** | slopeV500-1500 sma3nz amean y | 0.0027499349404465200 |
| **153** | slopeV500-1500 sma3nz stddevNorm y | 0.001061870894651890 |
| **154** | spectralFluxV sma3nz amean y | 0.0008930935172524510 |
| **155** | spectralFluxV sma3nz stddevNorm y | 0.002038569587764400 |
| **156** | mfcc1V sma3nz amean y | 0.0022500298782460100 |
| **157** | mfcc1V sma3nz stddevNorm y | 0.004207270457312170 |
| **158** | mfcc2V sma3nz amean y | 0.0018176815055178200 |
| **159** | mfcc2V sma3nz stddevNorm y | 0.004773431503866990 |
| **160** | mfcc3V sma3nz amean y | 0.002268856610491000 |
| **161** | mfcc3V sma3nz stddevNorm y | 0.004400338686907370 |
| **162** | mfcc4V sma3nz amean y | 0.002185621644811030 |
| **163** | mfcc4V sma3nz stddevNorm y | 0.08010847744002970 |
| **164** | alphaRatioUV sma3nz amean y | 0.0015859104438782900 |
| **165** | hammarbergIndexUV sma3nz amean y | 0.003807363710531110 |
| **166** | slopeUV0-500 sma3nz amean y | 0.0011988781086884300 |
| **167** | slopeUV500-1500 sma3nz amean y | 0.002218729661504880 |
| **168** | spectralFluxUV sma3nz amean y | 0.0040987423068144800 |
| **169** | loudnessPeaksPerSec y | 0.005526999288585810 |
| **170** | VoicedSegmentsPerSec y | 0.0025514308853237100 |
| **171** | MeanVoicedSegmentLengthSec y | 0.0013741533592871200 |
| **172** | StddevVoicedSegmentLengthSec y | 0.004486517941571810 |
| **173** | MeanUnvoicedSegmentLength y | 0.00135875465941385 |
| **174** | StddevUnvoicedSegmentLength y | 0.0028132528499626900 |
| **175** | equivalentSoundLevel dBp y | 0.0013693411520530100 |
| **176** | F0semitoneFrom27.5Hz sma3nz amean | 0.0027851533317420900 |
| **177** | F0semitoneFrom27.5Hz sma3nz stddevNorm | 0.0015016139610844800 |
| **178** | F0semitoneFrom27.5Hz sma3nz percentile20.0 | 0.0015907689094585400 |
| **179** | F0semitoneFrom27.5Hz sma3nz percentile50.0 | 0.0019559187727816600 |
| **180** | F0semitoneFrom27.5Hz sma3nz percentile80.0 | 0.002702483980237170 |
| **181** | F0semitoneFrom27.5Hz sma3nz pctlrange0-2 | 0.0017870476806484100 |
| **182** | F0semitoneFrom27.5Hz sma3nz meanRisingSlope | 0.002666047105469590 |
| **183** | F0semitoneFrom27.5Hz sma3nz stddevRisingSlope | 0.0019152764731080900 |
| **184** | F0semitoneFrom27.5Hz sma3nz meanFallingSlope | 0.0019239715112099900 |
| **185** | F0semitoneFrom27.5Hz sma3nz stddevFallingSlope | 0.0022961835254324100 |
| **186** | loudness sma3 amean | 0.0015710642867931700 |
| **187** | loudness sma3 stddevNorm | 0.007029474376764930 |
| **188** | loudness sma3 percentile20.0 | 0.002125492983691900 |
| **189** | loudness sma3 percentile50.0 | 0.0008268021797680990 |
| **190** | loudness sma3 percentile80.0 | 0.0007279185082038680 |
| **191** | loudness sma3 pctlrange0-2 | 0.00032943937605335700 |
| **192** | loudness sma3 meanRisingSlope | 0.0011550715007994600 |
| **193** | loudness sma3 stddevRisingSlope | 0.0030837785404793100 |
| **194** | loudness sma3 meanFallingSlope | 0.0007397604932363800 |
| **195** | loudness sma3 stddevFallingSlope | 0.002054191115104410 |
| **196** | spectralFlux sma3 amean | 0.001014200453954510 |
| **197** | spectralFlux sma3 stddevNorm | 0.0061089610867711700 |
| **198** | mfcc1 sma3 amean | 0.0034268892921921000 |
| **199** | mfcc1 sma3 stddevNorm | 0.005683253347886180 |
| **200** | mfcc2 sma3 amean | 0.001935755589284120 |
| **201** | mfcc2 sma3 stddevNorm | 0.02481273366589340 |
| **202** | mfcc3 sma3 amean | 0.003648959946783580 |
| **203** | mfcc3 sma3 stddevNorm | 0.012368366593167200 |
| **204** | mfcc4 sma3 amean | 0.00285539115365089 |
| **205** | mfcc4 sma3 stddevNorm | 0.030024735390912000 |
| **206** | jitterLocal sma3nz amean | 0.005881639312779950 |
| **207** | jitterLocal sma3nz stddevNorm | 0.0015666790829348900 |
| **208** | shimmerLocaldB sma3nz amean | 0.006464448679159240 |
| **209** | shimmerLocaldB sma3nz stddevNorm | 0.005450658424066750 |
| **210** | HNRdBACF sma3nz amean | 0.0018898643306895800 |
| **211** | HNRdBACF sma3nz stddevNorm | 0.023699621243737400 |
| **212** | logRelF0-H1-H2 sma3nz amean | 0.0017660440099732600 |
| **213** | logRelF0-H1-H2 sma3nz stddevNorm | 0.00916125897309504 |
| **214** | logRelF0-H1-A3 sma3nz amean | 0.0012895092702793400 |
| **215** | logRelF0-H1-A3 sma3nz stddevNorm | 0.0028306264840565600 |
| **216** | F1frequency sma3nz amean | 0.001384005465832510 |
| **217** | F1frequency sma3nz stddevNorm | 0.0027619763539588600 |
| **218** | F1bandwidth sma3nz amean | 0.004478647387887850 |
| **219** | F1bandwidth sma3nz stddevNorm | 0.0013347379099966000 |
| **220** | F1amplitudeLogRelF0 sma3nz amean | 0.0010034871846024200 |
| **221** | F1amplitudeLogRelF0 sma3nz stddevNorm | 0.0009926256592308800 |
| **222** | F2frequency sma3nz amean | 0.0014464716053864300 |
| **223** | F2frequency sma3nz stddevNorm | 0.0018646153828544200 |
| **224** | F2bandwidth sma3nz amean | 0.005447930172085440 |
| **225** | F2bandwidth sma3nz stddevNorm | 0.0016196516773530700 |
| **226** | F2amplitudeLogRelF0 sma3nz amean | 0.002255733828122440 |
| **227** | F2amplitudeLogRelF0 sma3nz stddevNorm | 0.0004357274287532970 |
| **228** | F3frequency sma3nz amean | 0.001106617133398530 |
| **229** | F3frequency sma3nz stddevNorm | 0.002337615196137140 |
| **230** | F3bandwidth sma3nz amean | 0.006932394508456960 |
| **231** | F3bandwidth sma3nz stddevNorm | 0.0013312195889272200 |
| **232** | F3amplitudeLogRelF0 sma3nz amean | 0.0007457934932484100 |
| **233** | F3amplitudeLogRelF0 sma3nz stddevNorm | 0.0018627270983087600 |
| **234** | alphaRatioV sma3nz amean | 0.0028060763996057900 |
| **235** | alphaRatioV sma3nz stddevNorm | 0.0064617458103709300 |
| **236** | hammarbergIndexV sma3nz amean | 0.0010213843783161200 |
| **237** | hammarbergIndexV sma3nz stddevNorm | 0.0019591548758221000 |
| **238** | slopeV0-500 sma3nz amean | 0.005135657834214870 |
| **239** | slopeV0-500 sma3nz stddevNorm | 0.013777135971375600 |
| **240** | slopeV500-1500 sma3nz amean | 0.014437876273915500 |
| **241** | slopeV500-1500 sma3nz stddevNorm | 0.007739852948153320 |
| **242** | spectralFluxV sma3nz amean | 0.0030185279538394600 |
| **243** | spectralFluxV sma3nz stddevNorm | 0.0023531805903663000 |
| **244** | mfcc1V sma3nz amean | 0.002838990453744620 |
| **245** | mfcc1V sma3nz stddevNorm | 0.00479040034255723 |
| **246** | mfcc2V sma3nz amean | 0.0012681510059961900 |
| **247** | mfcc2V sma3nz stddevNorm | 0.008178820552149640 |
| **248** | mfcc3V sma3nz amean | 0.004768159937710880 |
| **249** | mfcc3V sma3nz stddevNorm | 0.019091663387807300 |
| **250** | mfcc4V sma3nz amean | 0.0015512534962834400 |
| **251** | mfcc4V sma3nz stddevNorm | 0.0021870404425079300 |
| **252** | alphaRatioUV sma3nz amean | 0.003482159017702140 |
| **253** | hammarbergIndexUV sma3nz amean | 0.0004986639276406090 |
| **254** | slopeUV0-500 sma3nz amean | 0.0018575082432276600 |
| **255** | slopeUV500-1500 sma3nz amean | 0.002725678745671990 |
| **256** | spectralFluxUV sma3nz amean | 0.001898448643777710 |
| **257** | loudnessPeaksPerSec | 0.008192313797180260 |
| **258** | VoicedSegmentsPerSec | 0.0010209836238071800 |
| **259** | MeanVoicedSegmentLengthSec | 0.002596551364269080 |
| **260** | StddevVoicedSegmentLengthSec | 0.0030664382225700500 |
| **261** | MeanUnvoicedSegmentLength | 0.0023568216192807200 |
| **262** | StddevUnvoicedSegmentLength | 0.0027081819133325700 |
| **263** | equivalentSoundLevel dBp | 0.0017384426093570800 |

**Table S2.** Feature importance for TMT BTA. TMT BTA = Trail Making Test - processing time part A.

|  | **variable** | **importance** |
| --- | --- | --- |
| **0** | F0semitoneFrom27.5Hz sma3nz amean x | 0.0016342402337544000 |
| **1** | F0semitoneFrom27.5Hz sma3nz stddevNorm x | 0.000539910804322152 |
| **2** | F0semitoneFrom27.5Hz sma3nz percentile20.0 x | 0.0013780850456298900 |
| **3** | F0semitoneFrom27.5Hz sma3nz percentile50.0 x | 0.005607458478732280 |
| **4** | F0semitoneFrom27.5Hz sma3nz percentile80.0 x | 0.0024015185310682300 |
| **5** | F0semitoneFrom27.5Hz sma3nz pctlrange0-2 x | 0.0021638188345130200 |
| **6** | F0semitoneFrom27.5Hz sma3nz meanRisingSlope x | 0.003925265380233340 |
| **7** | F0semitoneFrom27.5Hz sma3nz stddevRisingSlope x | 0.0037864716799431200 |
| **8** | F0semitoneFrom27.5Hz sma3nz meanFallingSlope x | 0.003287611547593400 |
| **9** | F0semitoneFrom27.5Hz sma3nz stddevFallingSlope x | 0.0021545907457903600 |
| **10** | loudness sma3 amean x | 0.0019375657184399200 |
| **11** | loudness sma3 stddevNorm x | 0.001416762446827810 |
| **12** | loudness sma3 percentile20.0 x | 0.003345767084138190 |
| **13** | loudness sma3 percentile50.0 x | 0.004398960959638010 |
| **14** | loudness sma3 percentile80.0 x | 0.0012687792589490700 |
| **15** | loudness sma3 pctlrange0-2 x | 0.0004671098865862470 |
| **16** | loudness sma3 meanRisingSlope x | 0.0018377616634337000 |
| **17** | loudness sma3 stddevRisingSlope x | 0.004151298134496280 |
| **18** | loudness sma3 meanFallingSlope x | 0.0010482834749382800 |
| **19** | loudness sma3 stddevFallingSlope x | 0.0019918027381401400 |
| **20** | spectralFlux sma3 amean x | 0.0013275615831539200 |
| **21** | spectralFlux sma3 stddevNorm x | 0.0036373390475086700 |
| **22** | mfcc1 sma3 amean x | 0.002534004224721350 |
| **23** | mfcc1 sma3 stddevNorm x | 0.01082342895426570 |
| **24** | mfcc2 sma3 amean x | 0.0012638894418337800 |
| **25** | mfcc2 sma3 stddevNorm x | 0.006115974503472520 |
| **26** | mfcc3 sma3 amean x | 0.0022745715823126800 |
| **27** | mfcc3 sma3 stddevNorm x | 0.00617934545892492 |
| **28** | mfcc4 sma3 amean x | 0.0028243205824578300 |
| **29** | mfcc4 sma3 stddevNorm x | 0.005384964982259090 |
| **30** | jitterLocal sma3nz amean x | 0.004264759736645520 |
| **31** | jitterLocal sma3nz stddevNorm x | 0.000803428151248933 |
| **32** | shimmerLocaldB sma3nz amean x | 0.009124484836358200 |
| **33** | shimmerLocaldB sma3nz stddevNorm x | 0.0033270888345655300 |
| **34** | HNRdBACF sma3nz amean x | 0.0018262116886873300 |
| **35** | HNRdBACF sma3nz stddevNorm x | 0.0026238668415671500 |
| **36** | logRelF0-H1-H2 sma3nz amean x | 0.0027304290396715100 |
| **37** | logRelF0-H1-H2 sma3nz stddevNorm x | 0.0028194059522985600 |
| **38** | logRelF0-H1-A3 sma3nz amean x | 0.0009855018046824080 |
| **39** | logRelF0-H1-A3 sma3nz stddevNorm x | 0.001895553467132920 |
| **40** | F1frequency sma3nz amean x | 0.0007775322800881750 |
| **41** | F1frequency sma3nz stddevNorm x | 0.002450135786478060 |
| **42** | F1bandwidth sma3nz amean x | 0.0007806843999273890 |
| **43** | F1bandwidth sma3nz stddevNorm x | 0.0035470994528506800 |
| **44** | F1amplitudeLogRelF0 sma3nz amean x | 0.0007542762164626720 |
| **45** | F1amplitudeLogRelF0 sma3nz stddevNorm x | 0.0017606226407224600 |
| **46** | F2frequency sma3nz amean x | 0.0009312293811014660 |
| **47** | F2frequency sma3nz stddevNorm x | 0.0023498405114618400 |
| **48** | F2bandwidth sma3nz amean x | 0.0009674158635817140 |
| **49** | F2bandwidth sma3nz stddevNorm x | 0.0033245365554876300 |
| **50** | F2amplitudeLogRelF0 sma3nz amean x | 0.0003656983430796860 |
| **51** | F2amplitudeLogRelF0 sma3nz stddevNorm x | 0.0009629267303450070 |
| **52** | F3frequency sma3nz amean x | 0.0011931295801922800 |
| **53** | F3frequency sma3nz stddevNorm x | 0.0012083336996022200 |
| **54** | F3bandwidth sma3nz amean x | 0.0012688385296582400 |
| **55** | F3bandwidth sma3nz stddevNorm x | 0.003751721224998050 |
| **56** | F3amplitudeLogRelF0 sma3nz amean x | 0.0008121861137485580 |
| **57** | F3amplitudeLogRelF0 sma3nz stddevNorm x | 0.0014181080305298300 |
| **58** | alphaRatioV sma3nz amean x | 0.00026813485372536200 |
| **59** | alphaRatioV sma3nz stddevNorm x | 0.001175219797337180 |
| **60** | hammarbergIndexV sma3nz amean x | 0.00039610224746536800 |
| **61** | hammarbergIndexV sma3nz stddevNorm x | 0.001300393688246650 |
| **62** | slopeV0-500 sma3nz amean x | 0.0024846723422255500 |
| **63** | slopeV0-500 sma3nz stddevNorm x | 0.0022568609602305200 |
| **64** | slopeV500-1500 sma3nz amean x | 0.0016605178745046400 |
| **65** | slopeV500-1500 sma3nz stddevNorm x | 0.0054467333168976200 |
| **66** | spectralFluxV sma3nz amean x | 0.0005707262279271210 |
| **67** | spectralFluxV sma3nz stddevNorm x | 0.003371489665119850 |
| **68** | mfcc1V sma3nz amean x | 0.0010445640106959600 |
| **69** | mfcc1V sma3nz stddevNorm x | 0.001864503371004510 |
| **70** | mfcc2V sma3nz amean x | 0.003449849515899510 |
| **71** | mfcc2V sma3nz stddevNorm x | 0.0024640323569062900 |
| **72** | mfcc3V sma3nz amean x | 0.001980506632307420 |
| **73** | mfcc3V sma3nz stddevNorm x | 0.002973603175518520 |
| **74** | mfcc4V sma3nz amean x | 0.0015331406234470600 |
| **75** | mfcc4V sma3nz stddevNorm x | 0.02598466068450140 |
| **76** | alphaRatioUV sma3nz amean x | 0.0012238556014797700 |
| **77** | hammarbergIndexUV sma3nz amean x | 0.0021284318928727900 |
| **78** | slopeUV0-500 sma3nz amean x | 0.006828861560892870 |
| **79** | slopeUV500-1500 sma3nz amean x | 0.0013155796717402500 |
| **80** | spectralFluxUV sma3nz amean x | 0.023013167576834100 |
| **81** | loudnessPeaksPerSec x | 0.0022659492030267900 |
| **82** | VoicedSegmentsPerSec x | 0.010506989359799000 |
| **83** | MeanVoicedSegmentLengthSec x | 0.0007421933745358230 |
| **84** | StddevVoicedSegmentLengthSec x | 0.0013436635223421400 |
| **85** | MeanUnvoicedSegmentLength x | 0.0014861394581868000 |
| **86** | StddevUnvoicedSegmentLength x | 0.005088172761351160 |
| **87** | equivalentSoundLevel dBp x | 0.003084623611233290 |
| **88** | F0semitoneFrom27.5Hz sma3nz amean y | 0.000986469146177449 |
| **89** | F0semitoneFrom27.5Hz sma3nz stddevNorm y | 0.0016699975658256500 |
| **90** | F0semitoneFrom27.5Hz sma3nz percentile20.0 y | 0.0022314245765583100 |
| **91** | F0semitoneFrom27.5Hz sma3nz percentile50.0 y | 0.003723924646643430 |
| **92** | F0semitoneFrom27.5Hz sma3nz percentile80.0 y | 0.003494565043437740 |
| **93** | F0semitoneFrom27.5Hz sma3nz pctlrange0-2 y | 0.001366266842819110 |
| **94** | F0semitoneFrom27.5Hz sma3nz meanRisingSlope y | 0.004785306112870730 |
| **95** | F0semitoneFrom27.5Hz sma3nz stddevRisingSlope y | 0.0022501636905541800 |
| **96** | F0semitoneFrom27.5Hz sma3nz meanFallingSlope y | 0.002063013800377360 |
| **97** | F0semitoneFrom27.5Hz sma3nz stddevFallingSlope y | 0.01089990936418720 |
| **98** | loudness sma3 amean y | 0.004673530254257210 |
| **99** | loudness sma3 stddevNorm y | 0.0008026513603656020 |
| **100** | loudness sma3 percentile20.0 y | 0.0010107990194136800 |
| **101** | loudness sma3 percentile50.0 y | 0.0037628075654924000 |
| **102** | loudness sma3 percentile80.0 y | 0.0021830172349894400 |
| **103** | loudness sma3 pctlrange0-2 y | 0.0006860020291138900 |
| **104** | loudness sma3 meanRisingSlope y | 0.0009593940784691120 |
| **105** | loudness sma3 stddevRisingSlope y | 0.0008606615498514210 |
| **106** | loudness sma3 meanFallingSlope y | 0.0006592222086643840 |
| **107** | loudness sma3 stddevFallingSlope y | 0.0004861911428011750 |
| **108** | spectralFlux sma3 amean y | 0.0010907129637884800 |
| **109** | spectralFlux sma3 stddevNorm y | 0.002840376720974860 |
| **110** | mfcc1 sma3 amean y | 0.0004014718674311440 |
| **111** | mfcc1 sma3 stddevNorm y | 0.0005626167054009440 |
| **112** | mfcc2 sma3 amean y | 0.0023828434346420600 |
| **113** | mfcc2 sma3 stddevNorm y | 0.0053158253602102600 |
| **114** | mfcc3 sma3 amean y | 0.0025481462592805000 |
| **115** | mfcc3 sma3 stddevNorm y | 0.1264758847225380 |
| **116** | mfcc4 sma3 amean y | 0.0015969381794186100 |
| **117** | mfcc4 sma3 stddevNorm y | 0.0018367067479740000 |
| **118** | jitterLocal sma3nz amean y | 0.0026266346785133 |
| **119** | jitterLocal sma3nz stddevNorm y | 0.001697124515772440 |
| **120** | shimmerLocaldB sma3nz amean y | 0.001238020903538450 |
| **121** | shimmerLocaldB sma3nz stddevNorm y | 0.0009951295065602490 |
| **122** | HNRdBACF sma3nz amean y | 0.0009780069650975350 |
| **123** | HNRdBACF sma3nz stddevNorm y | 0.003242971628014910 |
| **124** | logRelF0-H1-H2 sma3nz amean y | 0.0021912674754083100 |
| **125** | logRelF0-H1-H2 sma3nz stddevNorm y | 0.004990014719405410 |
| **126** | logRelF0-H1-A3 sma3nz amean y | 0.0038274099667509200 |
| **127** | logRelF0-H1-A3 sma3nz stddevNorm y | 0.006113278745727930 |
| **128** | F1frequency sma3nz amean y | 0.0014181360630976000 |
| **129** | F1frequency sma3nz stddevNorm y | 0.005283854381279700 |
| **130** | F1bandwidth sma3nz amean y | 0.0016051701460223400 |
| **131** | F1bandwidth sma3nz stddevNorm y | 0.0023026830261730700 |
| **132** | F1amplitudeLogRelF0 sma3nz amean y | 0.0007203989429982480 |
| **133** | F1amplitudeLogRelF0 sma3nz stddevNorm y | 0.0012029111573924800 |
| **134** | F2frequency sma3nz amean y | 0.0009432115188426610 |
| **135** | F2frequency sma3nz stddevNorm y | 0.0018620996848406600 |
| **136** | F2bandwidth sma3nz amean y | 0.002955928006726570 |
| **137** | F2bandwidth sma3nz stddevNorm y | 0.00267969654145034 |
| **138** | F2amplitudeLogRelF0 sma3nz amean y | 0.0007066904199001110 |
| **139** | F2amplitudeLogRelF0 sma3nz stddevNorm y | 0.0011980631809115700 |
| **140** | F3frequency sma3nz amean y | 0.002327684300951370 |
| **141** | F3frequency sma3nz stddevNorm y | 0.0028770412432801900 |
| **142** | F3bandwidth sma3nz amean y | 0.0005054050438075930 |
| **143** | F3bandwidth sma3nz stddevNorm y | 0.0016683053300583800 |
| **144** | F3amplitudeLogRelF0 sma3nz amean y | 0.0018098379485536900 |
| **145** | F3amplitudeLogRelF0 sma3nz stddevNorm y | 0.0014359577989824700 |
| **146** | alphaRatioV sma3nz amean y | 0.0011717995017216200 |
| **147** | alphaRatioV sma3nz stddevNorm y | 0.0023013033416093900 |
| **148** | hammarbergIndexV sma3nz amean y | 0.001239105005044440 |
| **149** | hammarbergIndexV sma3nz stddevNorm y | 0.0014769061983626700 |
| **150** | slopeV0-500 sma3nz amean y | 0.0002324106217828160 |
| **151** | slopeV0-500 sma3nz stddevNorm y | 0.003184822299426920 |
| **152** | slopeV500-1500 sma3nz amean y | 0.0017708329910344500 |
| **153** | slopeV500-1500 sma3nz stddevNorm y | 0.002658377973340920 |
| **154** | spectralFluxV sma3nz amean y | 0.004789511131441500 |
| **155** | spectralFluxV sma3nz stddevNorm y | 0.0034008552983272100 |
| **156** | mfcc1V sma3nz amean y | 0.0033714205081542800 |
| **157** | mfcc1V sma3nz stddevNorm y | 0.0014622365303851500 |
| **158** | mfcc2V sma3nz amean y | 0.002889145289972100 |
| **159** | mfcc2V sma3nz stddevNorm y | 0.00655586128794564 |
| **160** | mfcc3V sma3nz amean y | 0.004137796743253760 |
| **161** | mfcc3V sma3nz stddevNorm y | 0.004098951027252860 |
| **162** | mfcc4V sma3nz amean y | 0.0013130255221792200 |
| **163** | mfcc4V sma3nz stddevNorm y | 0.0031999178803755500 |
| **164** | alphaRatioUV sma3nz amean y | 0.004544642451802230 |
| **165** | hammarbergIndexUV sma3nz amean y | 0.002049972054158650 |
| **166** | slopeUV0-500 sma3nz amean y | 0.0027984534255529400 |
| **167** | slopeUV500-1500 sma3nz amean y | 0.0016674582332384100 |
| **168** | spectralFluxUV sma3nz amean y | 0.0011755446102515500 |
| **169** | loudnessPeaksPerSec y | 0.011137467685018700 |
| **170** | VoicedSegmentsPerSec y | 0.0027232526626631300 |
| **171** | MeanVoicedSegmentLengthSec y | 0.002788746707594440 |
| **172** | StddevVoicedSegmentLengthSec y | 0.0019428215754908700 |
| **173** | MeanUnvoicedSegmentLength y | 0.0013628823485933000 |
| **174** | StddevUnvoicedSegmentLength y | 0.004071636833729200 |
| **175** | equivalentSoundLevel dBp y | 0.0028889600852041700 |
| **176** | F0semitoneFrom27.5Hz sma3nz amean | 0.0013884027080571400 |
| **177** | F0semitoneFrom27.5Hz sma3nz stddevNorm | 0.0027902061355373300 |
| **178** | F0semitoneFrom27.5Hz sma3nz percentile20.0 | 0.006168191013816500 |
| **179** | F0semitoneFrom27.5Hz sma3nz percentile50.0 | 0.001332518716382320 |
| **180** | F0semitoneFrom27.5Hz sma3nz percentile80.0 | 0.002539789417159200 |
| **181** | F0semitoneFrom27.5Hz sma3nz pctlrange0-2 | 0.0024681294434698600 |
| **182** | F0semitoneFrom27.5Hz sma3nz meanRisingSlope | 0.002611276303997970 |
| **183** | F0semitoneFrom27.5Hz sma3nz stddevRisingSlope | 0.0011497456171035300 |
| **184** | F0semitoneFrom27.5Hz sma3nz meanFallingSlope | 0.0028715232316614500 |
| **185** | F0semitoneFrom27.5Hz sma3nz stddevFallingSlope | 0.001262803240234940 |
| **186** | loudness sma3 amean | 0.0003455664264898200 |
| **187** | loudness sma3 stddevNorm | 0.008458958305903850 |
| **188** | loudness sma3 percentile20.0 | 0.0017904749530489300 |
| **189** | loudness sma3 percentile50.0 | 0.0012164132360065700 |
| **190** | loudness sma3 percentile80.0 | 0.0012747734134350000 |
| **191** | loudness sma3 pctlrange0-2 | 0.0014405716369390400 |
| **192** | loudness sma3 meanRisingSlope | 0.0017780682275444400 |
| **193** | loudness sma3 stddevRisingSlope | 0.0018702574098383500 |
| **194** | loudness sma3 meanFallingSlope | 0.0008378261506899150 |
| **195** | loudness sma3 stddevFallingSlope | 0.0014395020637752600 |
| **196** | spectralFlux sma3 amean | 0.0004085314146965150 |
| **197** | spectralFlux sma3 stddevNorm | 0.0029958354986034100 |
| **198** | mfcc1 sma3 amean | 0.0017200470578771900 |
| **199** | mfcc1 sma3 stddevNorm | 0.017860755124362100 |
| **200** | mfcc2 sma3 amean | 0.0020204473364765500 |
| **201** | mfcc2 sma3 stddevNorm | 0.023652350479233300 |
| **202** | mfcc3 sma3 amean | 0.003047208695890670 |
| **203** | mfcc3 sma3 stddevNorm | 0.002339315867941860 |
| **204** | mfcc4 sma3 amean | 0.00707725193152966 |
| **205** | mfcc4 sma3 stddevNorm | 0.007387948896330380 |
| **206** | jitterLocal sma3nz amean | 0.007756466026537570 |
| **207** | jitterLocal sma3nz stddevNorm | 0.002790955831332010 |
| **208** | shimmerLocaldB sma3nz amean | 0.002631161044095930 |
| **209** | shimmerLocaldB sma3nz stddevNorm | 0.0043272550683664100 |
| **210** | HNRdBACF sma3nz amean | 0.005912215605187900 |
| **211** | HNRdBACF sma3nz stddevNorm | 0.00831891160126199 |
| **212** | logRelF0-H1-H2 sma3nz amean | 0.0025404475226741400 |
| **213** | logRelF0-H1-H2 sma3nz stddevNorm | 0.0024339200702923100 |
| **214** | logRelF0-H1-A3 sma3nz amean | 0.0011537021599492400 |
| **215** | logRelF0-H1-A3 sma3nz stddevNorm | 0.0011686460835334600 |
| **216** | F1frequency sma3nz amean | 0.0031705787299609100 |
| **217** | F1frequency sma3nz stddevNorm | 0.005537191466725680 |
| **218** | F1bandwidth sma3nz amean | 0.0022701584834477700 |
| **219** | F1bandwidth sma3nz stddevNorm | 0.012494530745519600 |
| **220** | F1amplitudeLogRelF0 sma3nz amean | 0.0005660578450516130 |
| **221** | F1amplitudeLogRelF0 sma3nz stddevNorm | 0.0006504900810816290 |
| **222** | F2frequency sma3nz amean | 0.0016760984707256500 |
| **223** | F2frequency sma3nz stddevNorm | 0.0020971473629081400 |
| **224** | F2bandwidth sma3nz amean | 0.00221666666711406 |
| **225** | F2bandwidth sma3nz stddevNorm | 0.002621651637226580 |
| **226** | F2amplitudeLogRelF0 sma3nz amean | 0.002534955628397650 |
| **227** | F2amplitudeLogRelF0 sma3nz stddevNorm | 0.0024556204978655200 |
| **228** | F3frequency sma3nz amean | 0.002179935933542490 |
| **229** | F3frequency sma3nz stddevNorm | 0.0024645520744605000 |
| **230** | F3bandwidth sma3nz amean | 0.00200858628731303 |
| **231** | F3bandwidth sma3nz stddevNorm | 0.003415353908035110 |
| **232** | F3amplitudeLogRelF0 sma3nz amean | 0.00215764026906229 |
| **233** | F3amplitudeLogRelF0 sma3nz stddevNorm | 0.0017848844099250200 |
| **234** | alphaRatioV sma3nz amean | 0.012671393180794600 |
| **235** | alphaRatioV sma3nz stddevNorm | 0.023589315302715800 |
| **236** | hammarbergIndexV sma3nz amean | 0.011866328391945500 |
| **237** | hammarbergIndexV sma3nz stddevNorm | 0.012161758368824200 |
| **238** | slopeV0-500 sma3nz amean | 0.001302847141537030 |
| **239** | slopeV0-500 sma3nz stddevNorm | 0.004319907778160480 |
| **240** | slopeV500-1500 sma3nz amean | 0.004065077108509450 |
| **241** | slopeV500-1500 sma3nz stddevNorm | 0.004034605510980410 |
| **242** | spectralFluxV sma3nz amean | 0.0026892336726411100 |
| **243** | spectralFluxV sma3nz stddevNorm | 0.0028571344921581400 |
| **244** | mfcc1V sma3nz amean | 0.002560240802797870 |
| **245** | mfcc1V sma3nz stddevNorm | 0.03137804709352490 |
| **246** | mfcc2V sma3nz amean | 0.0038545648227332800 |
| **247** | mfcc2V sma3nz stddevNorm | 0.0016850945131511800 |
| **248** | mfcc3V sma3nz amean | 0.0027209952353813400 |
| **249** | mfcc3V sma3nz stddevNorm | 0.011993061910170600 |
| **250** | mfcc4V sma3nz amean | 0.0011464099900980900 |
| **251** | mfcc4V sma3nz stddevNorm | 0.005018464933888500 |
| **252** | alphaRatioUV sma3nz amean | 0.007057913191696620 |
| **253** | hammarbergIndexUV sma3nz amean | 0.0023982074833268800 |
| **254** | slopeUV0-500 sma3nz amean | 0.007260280913776930 |
| **255** | slopeUV500-1500 sma3nz amean | 0.0010710473050203500 |
| **256** | spectralFluxUV sma3nz amean | 0.0013264990373525700 |
| **257** | loudnessPeaksPerSec | 0.0061739613357153500 |
| **258** | VoicedSegmentsPerSec | 0.005757806078311130 |
| **259** | MeanVoicedSegmentLengthSec | 0.0022266392532219300 |
| **260** | StddevVoicedSegmentLengthSec | 0.004118683930125820 |
| **261** | MeanUnvoicedSegmentLength | 0.0014485128172888000 |
| **262** | StddevUnvoicedSegmentLength | 0.0014116199848798800 |
| **263** | equivalentSoundLevel dBp | 0.001151137454641420 |

**Table S3.** Feature importance for TMT BTB. TMT BTB = Trail Making Test - processing time part B.

**Appendix C**

|  | **T-statistic** | | **Pearson’s correlation** | | **Spearman’s correlation** | |
| --- | --- | --- | --- | --- | --- | --- |
|  | sex | | age | | education | |
| TMT BTA | *t* = -3.115 | *p* = 0.002 | *r* = 0.509 | *p* =  < 0.000 | *r* = -0.257 | *p* =  < 0.000 |
| TMT BTB | *t* = -1.918 | *p* = 0.056 | *r* = 0.460 | *p* =  < 0.000 | *r* = -0.269 | *p* =  < 0.000 |

**Table S4.** Relationship between the targets Trail Making Test - processing time part A (TMT BTA) and Trail Making Test - processing time part B (TMT BTB), and the confounding variables sex, age, and education.
